# In vitro assessment of the effect of magnetic fields on efficacy of biosynthesized selenium nanoparticles by *Alborzia kermanshahica*

**DOI:** 10.1186/s12896-024-00855-4

**Published:** 2024-05-09

**Authors:** Melika Eydelkhani, Shadi Kiabi, Bahareh Nowruzi

**Affiliations:** 1 Department of Biotechnology, Faculty of Converging Sciences and Technologies, Science and Research Branch, Islamic Azad University, Tehran, Iran; 2 Department of Biology, Tonekabon branch, Islamic Azad University, Tonekabon, Iran

**Keywords:** Cyanobacterial extract, Magnetic field, Selenium nanoparticles, Anticancer, HP-G2, *Alborzia Kermanshahica*

## Abstract

Cyanobacteria represent a rich resource of a wide array of unique bioactive compounds that are proving to be potent sources of anticancer drugs. Selenium nanoparticles (SeNPs) have shown an increasing potential as major therapeutic platforms and led to the production of higher levels of ROS that can present desirable anticancer properties. Chitosan–SeNPs have also presented antitumor properties against hepatic cancer cell lines, especially the Cht-NP (Chitosan–NPs), promoting ROS generation and mitochondria dysfunction. It is proposed that magnetic fields can add new dimensions to nanoparticle applications. Hence, in this study, the biosynthesis of SeNPs using *Alborzia kermanshahica* and chitosan (CS) as stabilizers has been developed. The SeNPs synthesis was performed at different cyanobacterial cultivation conditions, including control (without magnetic field) and magnetic fields of 30 mT and 60 mT. The SeNPs were characterized by uv-visible spectroscopy, Fourier-transform infrared spectroscopy (FT-IR), Dynamic light scattering (DLS), zeta potential, and TEM. In addition, the antibacterial activity, inhibition of bacterial growth, minimum inhibitory concentration (MIC), and minimum bactericidal concentration (MBC), as well as the antifungal activity and cytotoxicity of SeNPs, were performed. The results of uv-visible spectrometry, DLS, and zeta potential showed that 60 mT had the highest value regarding the adsorption, size, and stabilization in compared to the control. FTIR spectroscopy results showed consistent spectra, but the increased intensity of peaks indicates an increase in bond number after exposure to 30 mT and 60 mT. The results of the antibacterial activity and the inhibition zone diameter of synthesized nanoparticles showed that *Staphylococcus aureus* was more sensitive to nanoparticles produced under 60 mT. Se-NPs produced by *Alborzia kermanshahica* cultured under a 60 mT magnetic field exhibit potent antimicrobial and anticancer properties, making them a promising natural agent for use in the pharmaceutical and biomedical industries.

## Introduction

The advancements in nanotechnology have had a profound impact on various aspects of our lives, including bio-imaging and drug delivery in the field of medicine. Several methods have been employed to synthesize nanoparticles, including chemical, physical, and green chemistry approaches. The green synthesis approach has become increasingly popular due to its unique properties. It involves the use of microorganisms, plants, enzymes, or vitamins. The biological synthesis of nanoparticles is a reliable and cost-effective method that has shown cytotoxic effects on microorganisms and tumor cells. In addition, the process of synthesizing metal nanoparticles through chemical means results in the production of hazardous by-products, necessitates a significant amount of energy, and incurs substantial costs [1–7].

Selenium is an important element found in biological organisms and has various biological applications in fields such as health, biochemistry, genetics, and molecular biology [8]. These applications include formulations with antioxidant, antitumor, enzyme inhibitor, anti-infective, cytokine inducer, and immunomodulator properties [9–11]. The antibacterial activity of elemental selenium in nanoparticle form has been observed against S. *aureus*, a significant pathogen in hospital-acquired and medical device-associated infections. Some researchers have found that SeNPs are more effective and less toxic than silver nanoparticles [12].

Nanoparticles can be produced by a wide range of organisms, such as yeast, bacteria, fungi, plants, algae, and cyanobacteria [13]. Cyanobacteria are highly useful in the process of nanoparticle biosynthesis. This is because they possess natural chemicals that can effectively reduce metal ions, making them excellent reducing agents [14]. Algal species are highly proficient in nanoparticle formation thanks to their abundant proteins and enzymes. The process of biogenic synthesis using microalgae is a natural and environmentally friendly method that is cost-effective, non-toxic, and operates at low temperatures. It offers greater energy efficiency, ease of handling, and safety for a wide range of applications [15–18].

Algae and cyanobacteria possess rich sources of secondary metabolites with potential biotechnological applications in medicine. For example, two compounds, namely, meroterpene and usneoidone, have been isolated from *Cystophora* sp. and have also been reported to have antitumor properties [19–21]. Likewise, the sulfated polysaccharides of the brown alga *Eclonia cava* potently inhibited the growth of murine colon cancer (CT-26) and human leukemic monocytic lymphoma (U-937) cell lines [22].

In the last two decades, the cyanobacterial (microalgae) metabolites calothrixins A and B, ulithiacyclamide, borophycin, cryptophycin 1, largazole, apratoxin A, coibamide A, and curacin A have been reported to have dose-dependent antiproliferative and cytotoxic effects in various cancer lines, namely, human cancer cells, nasopharyngeal carcinoma cell lines and human epidermoid carcinoma, colorectal adenocarcinoma cell lines, colon adenocarcinoma, pancreatic ductal adenocarcinoma, breast adenocarcinoma, leukemia and Burkitt’s lymphoma cell lines [13, 23].

Microalgae production on a large-scale faces challenges due to high costs and water requirements. Strategies involving macro- and micronutrient manipulation and growing conditions can help reduce production costs and improve environmental sustainability [24]. Novel technologies, such as the use of magnetic fields (MFs), can enhance protein concentration and biomass production in microalgae cultivations [25]. Research is exploring new methods to improve the growth and production of biomolecules in microalgae. One such approach is the use of a magnetic field (MF) during the growing process [26]. MF has been shown to modify microbial metabolism, allowing algae to release more oxygen, accelerate photosynthesis, and facilitate metabolic activities, leading to faster growth. The effect of MF on microalgae depends on factors like cell health, exposure duration, strength, application, and the device used. Research indicates that MF exposure induces changes in free radical levels due to increased oxidative stress [27]. MF have been found to positively impact microalgae, with studies showing favorable outcomes in pigment content. The effects typically range from 10 to 250 mT. Various pigments, including chlorophyll a, chlorophyll b, carotenoids, and phycocyanin, have been observed on microalgae exposed to MF. Magnetic fields also increase protein content in microalgae. The use of magnetic treatments in spirulina cyanobacteria cultivation and its impact on phycocyanin pigment synthesis holds promise for a new approach in the pharmaceutical industry [28]. The non-toxic, pollution-free, affordable, and safe method of MF is promising for further research. However, it remains largely unexplored. While literature exists on its effects on biomass growth and products, there is a lack of research on MF’s impact on the antibacterial, antioxidant, antifungal, and anticoagulant properties of pure pigments, especially in relation to cyanobacterial species. This lack of resources limits the available resources.

Since many cyanobacterial strains have been isolated from metal and metalloid polluted aquatic environment and are ubiquitous organisms, they are an excellent model organism to study toxicological effects of metals and metalloids and also serve as a tool to bioremediate them. Furthermore, cyanobacteria are also capable of synthesizing metal nanoparticles (MNPs) when exposed to high concentrations of metals viz. Ag, Au, Pd, Pt and Zn as one of the resistance mechanisms involving reduction of metal ions [29–31]. Although there are several reports on bacteria-mediated biosynthesis and characterization of SeNPs [32, 33], very few reports have elucidated cyanobacteria-mediated SeNPs biosynthesis, characterization in conjugation with magnetic field as a cancer treatment method. These potential cyanobacterial strains are *Anacystis nidulans*, *Anabaena variabilis* [34], *Phormidium luridum var. olivacea* [35], and *Spirulina platensis* [36, 37].

Green nanoparticles have been reported to prevent and inhibit malignant cell progression, with *Nostoc* sp. HKAR-2 synthesizing SNPs showing significant cytotoxicity against MCF-7 [38]. To combat the harmful effects of anticancer therapies, modern technological applications using natural materials, such as bio-nanoparticles from cyanobacteria, are being explored.

Chitosan (CS) is a linear, biodegradable polysaccharide rich in the exoskeleton of crustaceans. Chitosan nanoparticles (Ct-NP) are ideal carriers of bioactive and therapeutic compounds such as quercetin, venlafaxine hydrochloride and oligonucleotides [39]. Of note, the nature of positive charges in acidic solutions allows Chitosan to target cells with negatively charged microenvironment, a feature intrinsic to some cancers. Conjugation of selenite or selenic group (–SeO3) to Chitosan has been shown to promote cell death in human sarcoma and leukemia K562 cells [40, 41].

New Genus *Alborzia kermanshahica* was, originally described by Nowruzi & Soares (2022) from Kermanshah province (Iran), belongs to order Chroococcales and family Chroococcaceae. Nowruzi et al., 2022, recoded the presence of a gene cluster coding for the biosynthesis of a bioactive compound that is very rare in this family and present of toxic compounds (microcystin) which might account for the poisoning of human [42]. Considering the presence of microcystin toxin in *Alborzia kermanshahica* in this study, the anticancer activity of nanoparticles synthesized by *Alborzia kermanshahica* using the magnetic fields as a novel strategy was investigated. As magnetic fields can add new dimensions to nanoparticle applications. In addition, since no study has been conducted on the effect of magnetic fields on the production of selenium nanoparticles and their anti-cancer effects, the results of this study can be considered an important step in the introduction of new anti-cancer compounds.

## Materials and methods

### Materials

All chemicals and other analytical components were purchased from Hi-Media, Merck, and Sigma, respectively. Double-distilled water was used in the production of all reagents and buffers.

### Culture conditions of the cyanobacterial strain

Cyanobacterial strain *Alborzia kermanshahica* isolated from Cyanobacteria culture collection (CCC) of herbarium ALBORZ at the Science and Research Branch, Islamic Azad University, Tehran and was cultured in an Erlenmeyer flask containing 100 mL of liquid medium, with pH adjusted to 7.2. Cultures were then maintained at 28 ± 2 ºC with periodic shaking, under a photoperiod of 14:10 h (light: dark) cycle and illuminated with ca. 50–55 µmol photons m^− 2^ s-1.

### Application of magnetic field on the culture of cyanobacteria *Alborzia kermanshahica*

A study of static magnetic field (SMF) in assays with *Alborzia kermanshahica* was carried out with ferrite magnets and electric current (in solenoid) that were applied around VTP. Each magnet was at 180 º, 15 cm above the base of the VTP. *Two different models of magnetic magnets* with a mean intensity of 30 mT (150 × 50 × 10 mm) and 60 mT (50 × 50 × 25 mm) were applied to the cyanobacterial cells for 21 days, and then the cells were centrifuged and collected (g x 5000). Both intensities (30 and 60 mT) were measured in the center of the VTP by a Globalmag MF measuring device (model TLMP-HALL-05k-T0, Brazil). Then, the effects of magnetic fields were compared with control cultures [43, 44].

### SeNPs biosynthesis

A cryogenically preserved 1 gram of powdered biomass, which was cultured under a magnetic field, was utilized to create a 1-liter aqueous extract. This was achieved by placing the mixture in a water bath for 10 min at 60 °C. Following this, the mixture was subjected to centrifugation at 6000 rpm for 15 min at a specific temperature. The resulting supernatant was then collected and filtered using a Whatman filter No. 1. The reaction mixture, consisting of 1 mM sodium selenite and extract in a 1:2 ratio, was incubated at 32 °C for the synthesis of SeNPs. A control solution of sodium selenite (1 mM) without extract was also maintained under identical conditions. The solution color changed upon the observation of synthesized SeNPs. Following the reaction, the solution containing nanoparticles underwent centrifugation at 15,000 rpm for 20 min at 4 °C. The pellet was washed three times by centrifuging with double-distilled water to remove any remaining substances [45, 46].

### Stabilization and coating of selenium nanoparticles with chitosan

Chemical reduction of cobalt selenite was used to produce selenium nanoparticles that were stabilized with chitosan of high molecular mass. The cross-linker used for Cht-NP (Chitosan–NPs) synthesis was sodium tripolyphosphate (TPP; Sigma-Aldrich, St. Louis, MO, USA). Preparations were made of a 0.1% stock solution (in acetic acidified solution) and a 0.5% sodium tripolyphosphate )TPP( solution (in DIW) pH = 5.2). The Cht solution was stirred vigorously, while the TPP solution was added slowly to it using a syringe needle at a rate of 0.3 mL/min. The opalescent suspension of formed Cht-NPs was stirred for an additional 115 min. After that, the NP pellet was collected by centrifugation at a speed of 10,500 × g for 30 min and washed repeatedly with DIW [47].

### Characterization of AgNPs

#### UV-Visible spectroscopy

After 24 h, a portion of the bio fabricated selenium nanoparticle was taken and analyzed using uv-visible spectroscopy. The analysis was conducted using a UV1800 PC spectrophotometer from Shimadzu, Japan, with a wavelength range of 300 nm to 700 nm and a resolution of 1 nm.

#### Fourier-transform infrared spectroscopy (FTIR)

The functional groups and composition of SeNPs were analyzed using FTIR spectroscopy (Shimadzu, Japan). The SeNPs powders were mixed in potassium bromide at a ratio of 1:100. The FT-IR instrument was operated in diffuse reflectance mode, specifically DRS-800. The spectra were collected over a range of 400 to 4000 cm − 1 with a resolution of 4 cm − 1. The identification of functional groups was accomplished by utilizing reference spectra [48].

#### DLS and zeta potential

The size of silver nanoparticles synthesized by cyanobacterial strains was measured. Samples with specific absorption of selenium nanoparticles, which were free of settling and had colloidal stability, were selected. Four glass vials containing 5 milliliters of the prepared supernatant with SeNPs were set up. The glass vials were covered with aluminum foil to prevent light penetration and particle agglomeration. DLS and zeta potential analysis were performed to assess light scattering and investigate the zeta potential. For this purpose, the Malvern Zetasizer Nano ZS® instrument was used. The measurement parameters included a laser with a wavelength of 633 nm (He–Ne), a constant scattering angle of 173°, a measurement temperature of 25 °C, a medium viscosity of 0.8872 mPa⋅s, a medium refractive index of 1.330, and a material refractive index of 1.59. Prior to DLS measurement, the colloid underwent filtration through a 0.2 μm polyvinylidene fluoride (PVDF) membrane. Subsequently, the sample was introduced into a quartz microcuvette [49, 50].

### Transmission electron microscopy (TEM)

The impact of nanoparticles on *Escherichia coli* bactericidal was analyzed using TEM. Samples were placed on a grid made of copper coated with carbon (200-mesh) and examined with the Tecnai G2 20 microscope (FEI, Amsterdam, The Netherlands) [45, 48].

### Antibacterial efficiency of Se-NPs

The antimicrobial effectiveness of the synthesized SeNPs was assessed using the disk diffusion method against indicator strains of *Staphylococcus aureus* and *Escherichia coli*. The Kirby-Bauer disk susceptibility test was conducted using standardized procedures. Each petridis was inoculated with 100 µl of a 0.5 McFarland turbidity bacterial suspension (approximately 2 × 108 cfu/ml) onto nutrient agar plates. The bacterial suspension was evenly spread using a sterile swab. Prepared discs containing SeNPs nanoparticle at a concentration of 20 µg/disc were placed on the inoculated plates at the correct spacing. Then, the plates incubated for 16 h at a temperature of 37 °C. Ultimately, the measurement of the expansion of the inhibition zone around the loaded discs was recorded in millimeters [45].

### Inhibition zone diameter of bacterial growth

The optical density (OD) in the liquid medium of MIC tubes was determined using uv-vis spectroscopy at 600 nm to determine the inhibition of bacterial growth. The measurements were taken at a concentration range of 0 to 200 ppm. Reduced absorption resulted in a decrease in bacterial growth. The control sample used in this test was identical to the control sample employed in the MIC test. The antibacterial effects of each dose of synthesized nanoparticles were quantified as the percentage of bacterial growth inhibition, as calculated by the provided Eq. [51]:

$${\rm{P}}\,\left( {{\rm{GI}}\% } \right)\,{\rm{ = }}\,{\rm{1 - }}\,{\rm{OD}}\,{\rm{sample/OD}}\,{\rm{control}}\, \times \,{\rm{100}}$$

$${\rm{Growth}}\,{\rm{Inhibit}}\,{\rm{Percentage}}\,\left( {{\rm{GI\% }}} \right)\,{\rm{ = }}\,{\rm{1 - }}\frac{{OD{\rm{ }}sample}}{{OD{\rm{ }}blank}}\,{\rm{ \times }}\,{\rm{100}}$$

### MIC and MBC determination of Se-NPs

The microtiter broth-dilution method was employed to determine the minimum inhibitory concentration (MIC) and minimum bactericidal concentration (MBC) of Se-NPs. The minimum inhibitory concentration (MIC) was determined by introducing the indicator organisms into a set of wells containing varying concentrations of the synthesized SeNPs, ranging from 1000 to 125 ppm, with two-fold dilutions. Following a standardized incubation period, the MIC is determined as the minimum concentration of the antimicrobial agent that effectively inhibits the growth of the organism. Following the MIC determination of the SeNPs, small amounts (0.01 mL) from all tubes that exhibited no observable bacterial growth were placed onto nutrient agar plates. Organisms that were inhibited but not killed in the MIC test now have an opportunity to grow due to a significant dilution of SeNPs. The MBC is determined as the minimum concentration of the antimicrobial agent that reduces the number of colonies by 99.9% after a standard incubation period. The determinations were conducted in triplicate.

### Antifungal assay

The antifungal activities of SeNPs against *Aspergillus niger* and *Penicillium chrysogenum* were determined by using the National Committee for Laboratory Standards (CSLI) for yeasts (CSLI M27-A) and filamentous fungi (CSLI M38-P) approved for both macro- and microdilution methods [52, 53]. *Aspergillus niger* and *Penicillium chrysogenum* were cultured at 25 °C in Sabouraud’s dextrose agar medium, respectively. Preparations of 0.5–2.5 × 103 and 2–5 × 104 conidia/mL (final concentration) were obtained from *Aspergillus niger* and *Penicillium chrysogenum*, respectively, by using a standard neo bar slide with the tubes containing 3 mL of sterile saline solution. RPMI 1640 medium containing l-glutamine and phenol red was buffered to pH 7.0 at 25 °C with 0.165 mol/L 4-morpholinepropanesulfonic acid and used as the MIC method. SeNPs were suspended in sterile distilled water and subsequently diluted to a final concentration of 10–200 µg/mL with the medium according to the CLSI standard. The MICs of SeNPs capable of fungal growth inhibition after 48 h of incubation at 37 °C were determined. RPMI medium containing fungal suspension without SeNPs was used as negative control. Amphotericin B (AmB) (10 µg/mL) and nystatin (15 µg/mL) were used in the same medium as positive controls for *Aspergillus niger* and *Penicillium chrysogenum*, respectively. All antifungal experiments were repeated three times.

### Cytotoxicity assay

The in vitro cytotoxicity tests of the synthetized nanoparticles were performed on the viability of HepG-2 cell lines. Cells viability was measured after 24 h and 48 h of incubation with different doses of nanoparticles. Briefly, 100 µl containing approximately 1.5 × 10^5^ cells were seeded into each well containing 1 mL of minimum essential media (MEM, Gibco), and incubated at 20 °C for 24 h. Following reaching a monolayer, all wells were inoculated with new media containing four different concentrations of SeNPs (0-200 ppm), except for the negative control wells, and incubated at 20℃ for 24 h. The selected concentrations were obtained based on MIC values. Cytotoxicity of selected nanoparticle doses was measured by trypan blue exclusion assay [54]. In brief, after incubation periods, the culture medium was decanted, and the cells were gently washed with phosphate-buffered saline to eliminate the remaining nanoparticles. The cells were mixed (1:1) with 0.4% trypan blue for 60 s. The dye was fixed with 4% formalin for 10 min and then the cells were washed three times with phosphate-buffered saline and examined under an inverted microscope. The number of dead cells (blue) was counted against the number of live cells (unstained) per hundred cells. The percentage of viable cells per well was expressed as the mean ± SE. The test was performed in three replicates with three replicates of each well and one control well per assay [55].

### Statistical analysis

Statistical evaluation of the results was made with SPSS 16.0 (SPSS Inc. Chicago, Illinois, USA). All values were expressed as mean ± SEM. The data were statistically analyzed by one-way analysis of variance (ANOVA). In view of the exploratory nature of the study, probability values *P* ≤ 0.05 were regarded as statistically significant.

## Results

### Optical analysis by UV-Visible spectroscopy

**Fig. 1:**
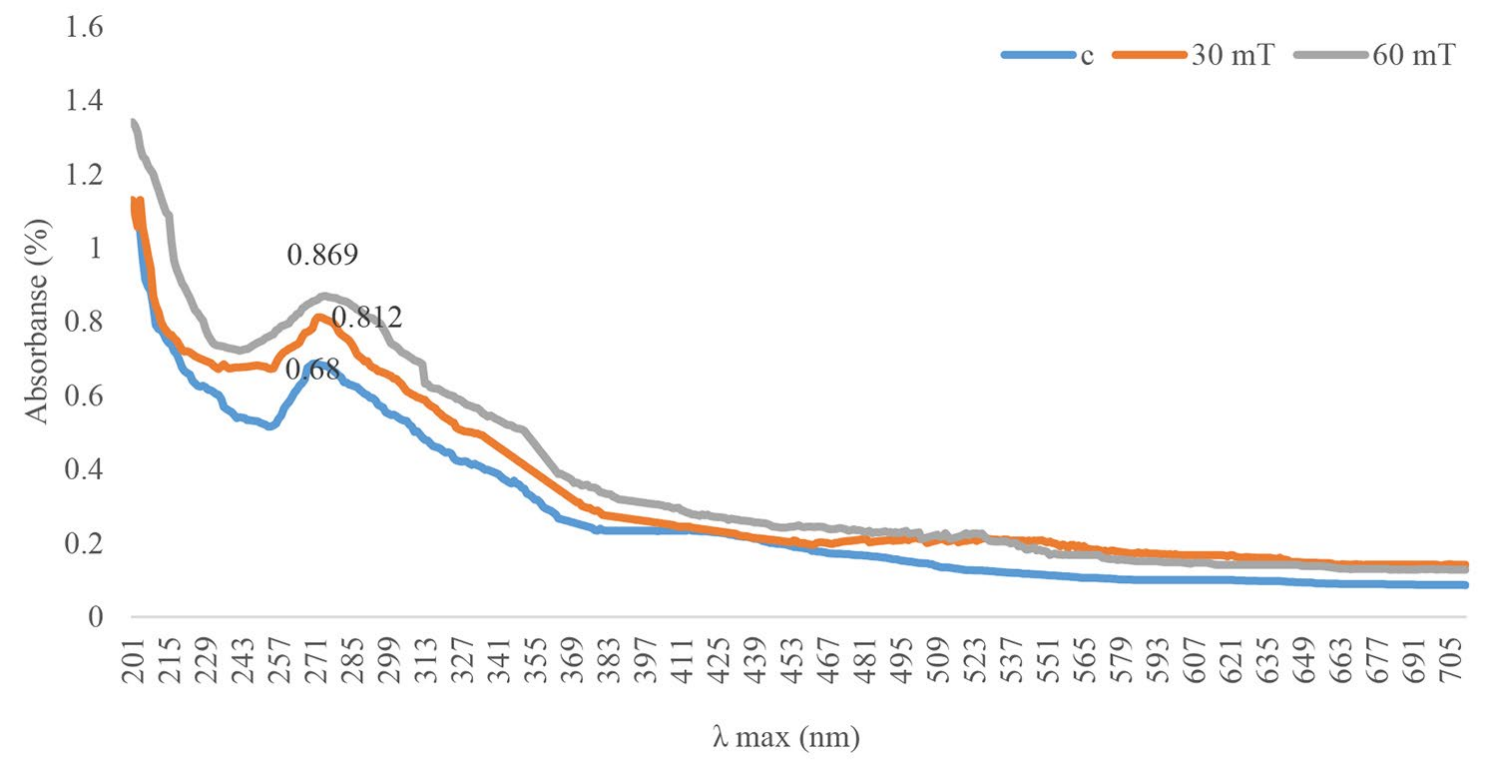
Spectroscopy diagrams of selenium nanoparticles prepared *from Alborzia kermanshah*ica cyanobacteria cultivated in three conditions

The results of the comparative uv-visible absorption spectra of selenium nanoparticles biosynthesized by *Alborzia kermanshah*ica are presented in Fig. 1. In Fig. 1, the formation of nanoparticles was shown. The ʺmax of selenium nanoparticles from cyanobacteria grown in their normal environment was 269 nm. The ƛmax of selenium nanoparticles prepared from the extract of cyanobacteria grown by applying a magnetic field of 30 mT and 60 mT were observed at wavelengths of 272 nm and 273 nm, respectively. The results of uv-vis spectrometry showed that 60 mT had the highest adsorption.

### FT-IR analysis

**Fig. 2:**
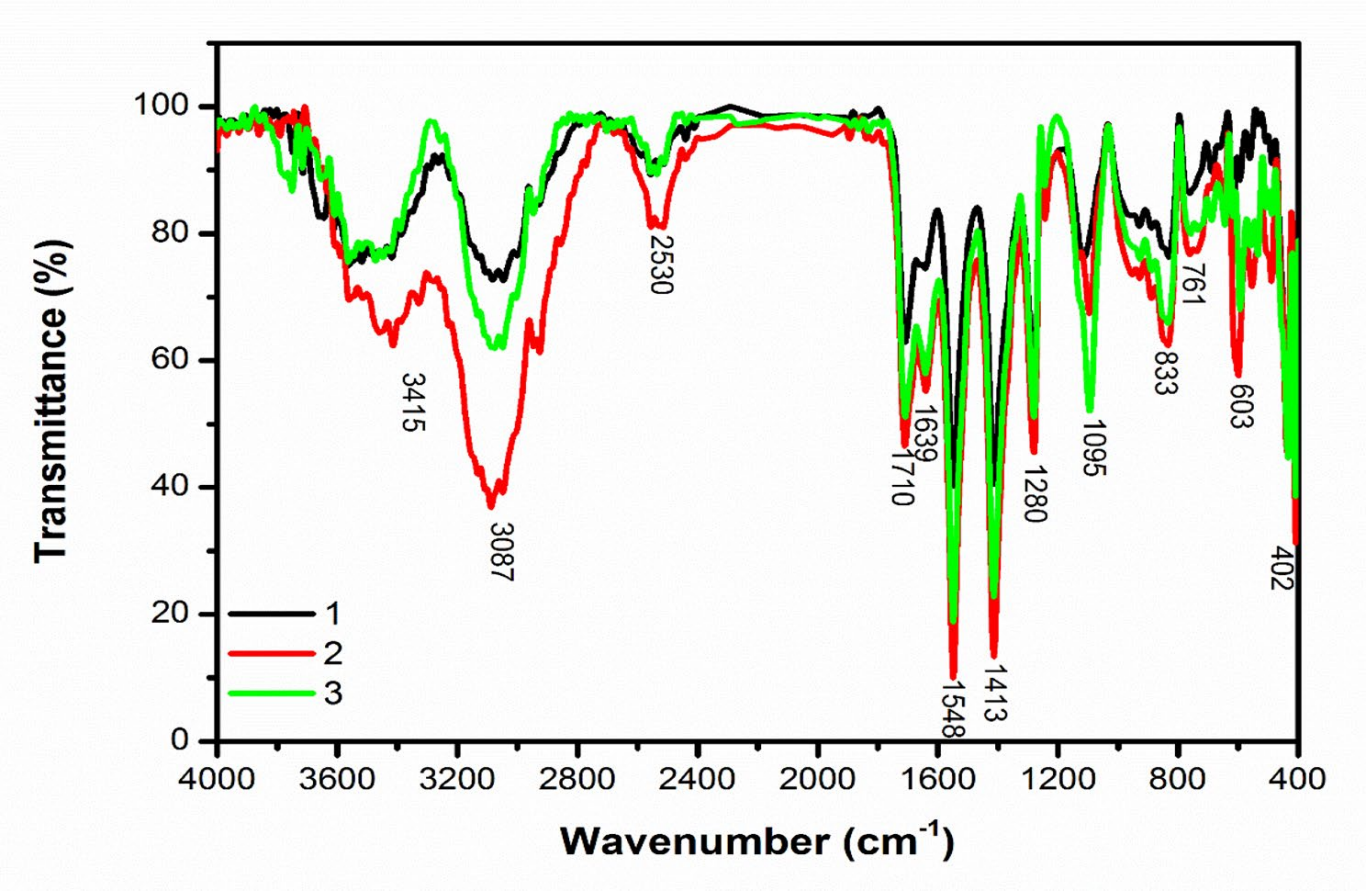
FTIR diagrams of selenium nanoparticles prepared *from Alborzia kermanshah*ica cyanobacteria cultivated in three conditions of [1] control [2], 30 mT and [3] 60 mT

FT-IR analysis was used to investigate the chemical bonds present in the samples under study. Figure 2 depicts the FT-IR spectrum of the examined sample.

In the spectra related to these three substances, the characteristic peaks appearing at the wavelengths of 3087 cm-1 and 3415 cm-1 are related to the stretching vibrations of N-H and O-H bonds in the amine and hydroxyl structures in the structure of cyanobacteria and chitosan, respectively. Also, the peak located at 2530 cm-1 wave number is related to the stretching vibrations of S-H bonds in the thiol structure in cyanobacteria.

The peak appearing at 1710 cm-1 wave number is related to the stretching vibration of C = O bonds in carboxyl structures in cyanobacteria and chitosan compounds. Also, the peaks located at the wavelengths of 1639 cm-1 and 1548 cm-1 are respectively related to the bending vibrations of O-H bonds in hydroxyl structures and the bending vibration of N-H bonds in amide compounds present in compounds adsorbed on the surface of selenium nanoparticles.

The peaks located at 1413 cm-1, 1280 cm-1, 1095 cm-1 and 837 cm-1 wavenumbers are related to the bending vibration of C-H bonds in methyl and methylene structures, the stretching vibration of C- OH and C-O-C bonds, and the dancing vibration of the C-H bond in the extract compounds. The peak appearing in the wave number cm-1 761 is related to the bending vibration of the aromatic rings in the structure of the material compounds.

The peak appearing at the wave number 603 cm-1 is related to the rocking vibration of C-H bonds in the aliphatic structures found in the organic compounds in this material. Also, in this spectrum, a strong and sharp peak at the wave number cm-1 402 has appeared, which is related to the stretching vibration of the Se-O bond in the selenium oxide structure and confirms the formation of these nanoparticles.

By comparing the intensity of the peaks related to these three samples, it is clear that the nanoparticles produced by the magnetic field increased the intensity of the peaks, which proves the increase in the number of bonds related to each peak after being subjected to the magnetic field. It is also clear that the intensity of the peaks in sample 2, in which selenium nanoparticles synthesized with the help of cyanobacteria and coated with chitosan and treated in a magnetic field of 30 mT, had the highest intensity among the investigated samples. The results showed that 30 mT magnetic field can be considered as the optimal condition to obtain a sample with the highest leveling modification factors (Table 1).

**Table 1:** Functional groups identified in different wave numbers of synthesized SeNPs

Functional groups	Control	30 mT	60 mT
N-H stretching vibrations	3087.526	3087.526	3087.526
O-H stretching vibrations	3415.371	3415.371	3415.371
S-H stretching vibrations	2530.19	2530.19	2530.19
C = O stretching vibrations	1710.578	1710.578	1710.578
O-H Bending vibration	1639.224	1639.224	1639.224
N-H Bending vibration	1548.584	1548.584	1548.584
C-H Bending vibration	1413.589	1413.589	1413.589
C-OH stretching vibrations	1280.523	1280.523	1280.523
C-O-C stretching vibrations	1095.387	1095.387	1095.387
Rocking vibration of C-H	836.9684	836.9684	836.9684
Bending vibration of aromatic rings	761.7569	761.7569	761.7569
C-H rocking vibration	603.6201	603.6201	603.6201
Se-O stretching vibrations	401.1277	401.1277	401.1277

### DLS results

**Fig. 3:**
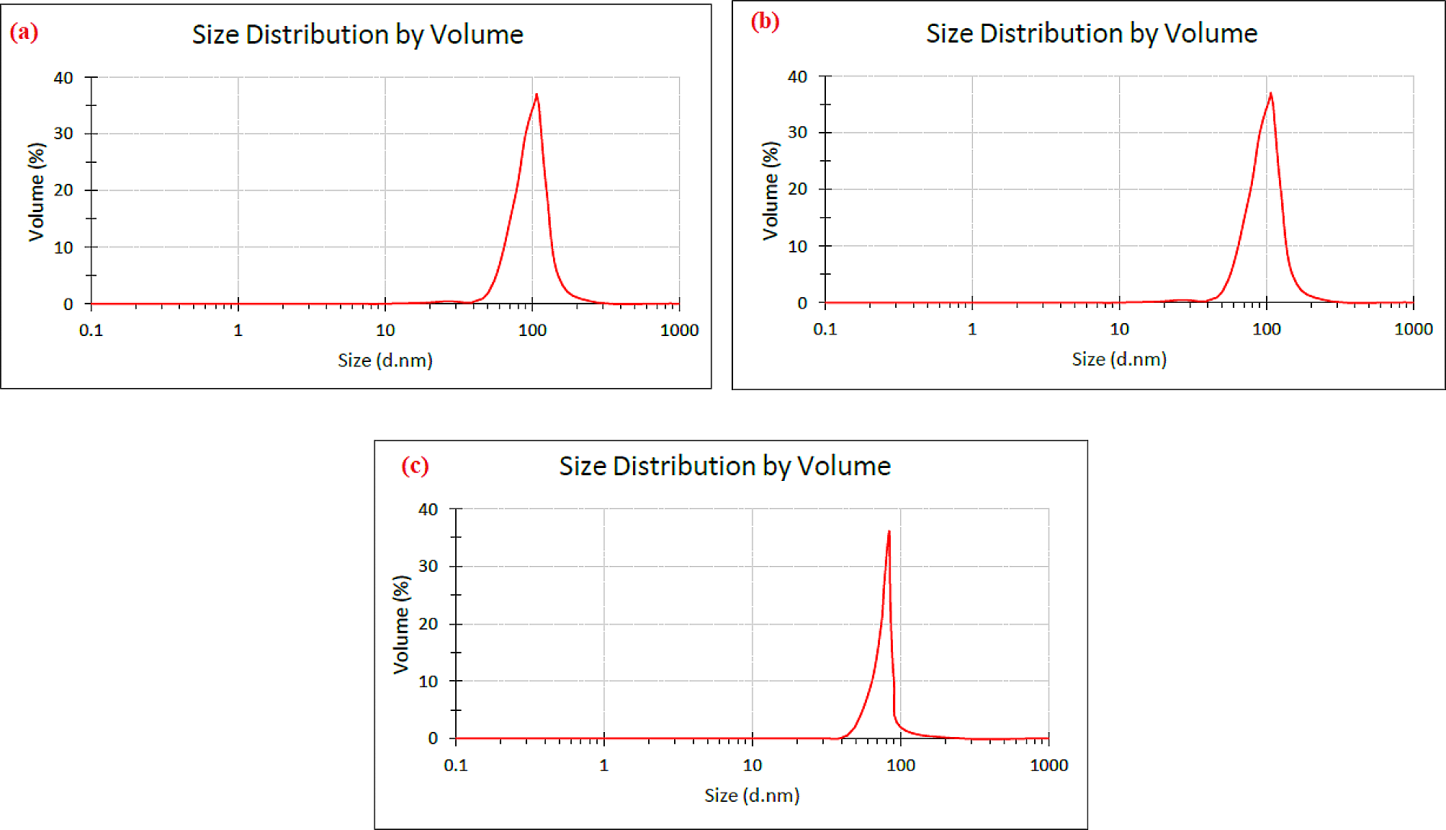
DLS to selenium nanoparticles synthesized with *Alborzia kermanshahica* cyanobacterial extract cultivated in three conditions (**a**): control / (**b**): 30mT / (**c**): 60mT

Examining the results of selenium nanoparticles obtained from cyanobacterial extract grown in control conditions showed a single peak curve with a width of 62 nm (Fig. 3-a). The results showed the uniformity of the droplet size distribution in all samples, and the polydispersity index value was reported as 0.422. Also, 52.3% of the nanoparticles had a size equal to 105.7 nm. The results of selenium nanoparticles obtained from the extract of cyanobacteria grown in 30 mT conditions showed a single peak curve with a width of 31 nm, and the value of the polydispersity index was reported as 0.392 (Fig. 3-b), and 61.9% of the nanoparticles had a size equal to 105.7 nm.

The results of selenium nanoparticles obtained from cyanobacterial extract grown in mT 60 conditions showed a single peak curve with a width of 21 nm, and the value of the polydispersity index was reported as 0.338 (Fig. 3-c). Also, 45.5% of the nanoparticles had a size equal to 87.7 nm.

### Zeta potential analysis

**Fig. 4:**
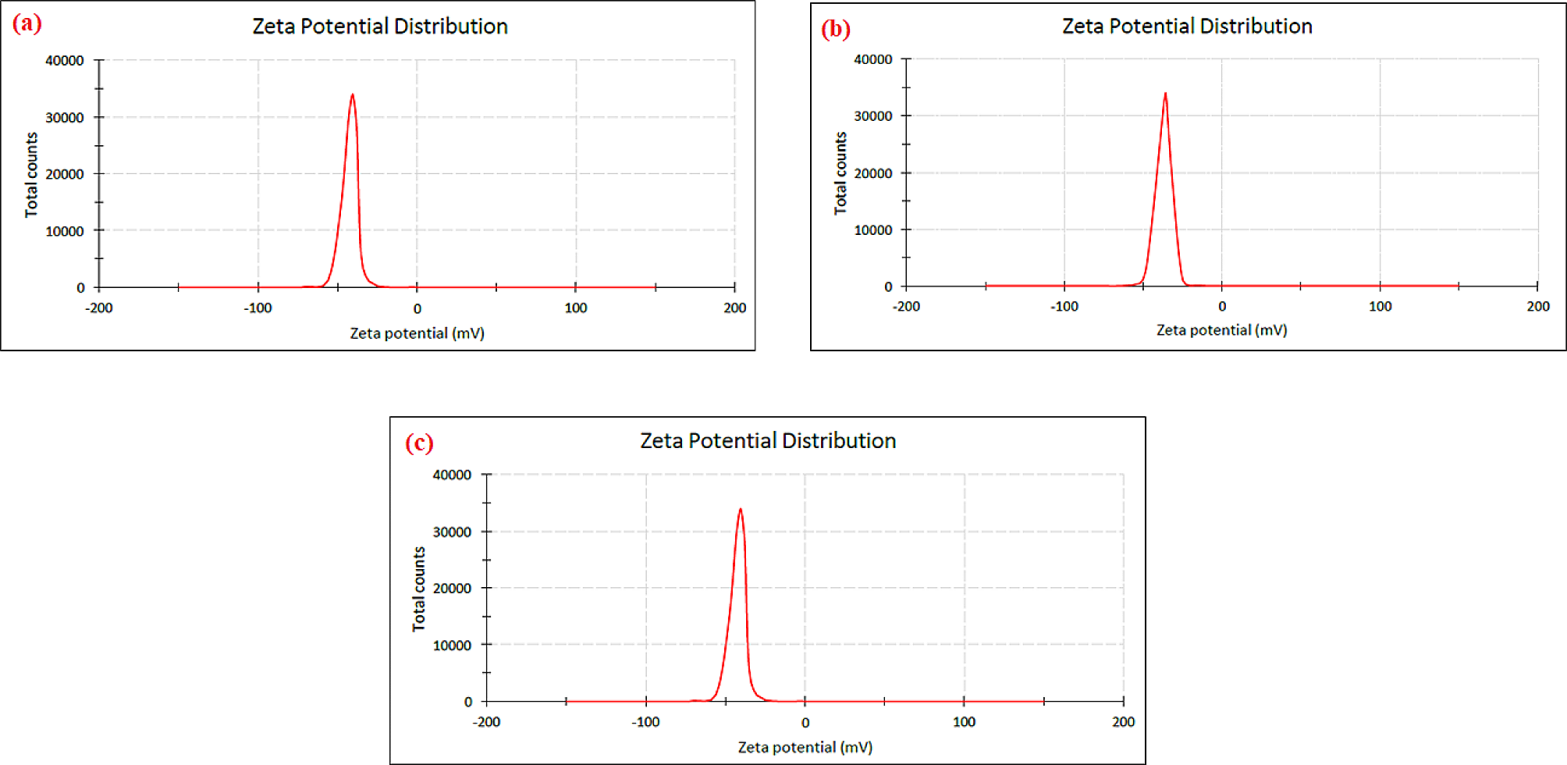
Zeta potential to selenium nanoparticles synthesized with *Alborzia kermanshahica* cyanobacterial extract cultivated in three conditions (**a**): control / (**b**): 30mT / (**c**): 60mT

The results of the zeta potential diagram showed that the zeta potential of the nanoparticles produced from the extract of cyanobacterium *Alborzia kermanshahica* cultivated in control conditions was equal to -1.28 mV with a width of 6 mV, and the particles had a conductivity of 0.219 mS/cm (Fig. 4-a). The zeta potential of nanoparticles under 30 mT conditions was equal to -36.7 mV with a width of 4.5 mV, and the conductivity of the particles was equal to 0.326 mS/cm (Fig. 4-b). The zeta potential of nanoparticles under 60 mT conditions was equal to -40.5 mV with a width of 5.2 mV, and the conductivity of the particles was equal to 0.262 mS/cm (Fig. 4-c).

### Antibacterial activity of Selenium nanoparticles

#### Inhibition zone diameter

The inhibition zone diameter of nanoparticles in control conditions and the application of magnetic intensities of 30 and 60 mT are shown in Table 2. The results of the inhibition zone diameter of nanoparticles under control conditions and applying magnetic fields of 30 and 60 mT showed that no significant difference was observed between the different treatments (*p* > 0.05). However, in the comparison between the two investigated strains, the *Staphylococcus aureus* strain was more sensitive than *Escherichia coli* (*p* > 0.05).

**Table 2:** Inhibition zone diameter of selenium nanoparticles (mm)

	Control	30 mT	60 mT
*Staphylococcus aureus*	8.33 ± 0.47 ^b^	8.66 ± 0.47 ^ab^	9.00 ± 0.81 ^a^
*Escherichia coli*	6.66 ± 0.00 ^b^	7.33 ± 0.00 ^ab^	7.66 ± 0.00 ^a^

• Different lowercase letters indicate significant differences in each row (*P* < 0.05)

#### MIC and MBC results

*Staphylococcus aureus* and *Escherichia coli*.

The results of the MIC and MBC of nanoparticles produced under control conditions and magnetic fields of 30 and 60 mT are presented in Table 3. The results of MIC and MBC showed that the application of both magnetic fields had the same inhibitory effect on *Staphylococcus aureus* and *Escherichia coli* compared to the control sample. In the comparison between the two groups of bacteria, *Escherichia coli* grew at a higher concentration of the obtained extracts for inhibition (*p* < 0.05). Also, no significant difference was observed between the magnetic field of 30 and 60 mT in the MIC of *Escherichia coli* bacteria (*p* > 0.05), while the lowest concentration of MBC was reported in Staphylococcus *aureus* and *Escherichia coli* under magnetic conditions of 60 mT (*p* < 0.05).

**Table 3:** selenium nanoparticles MIC & MBC

Pathogens	MIC (mg/ml)		
	Co	30mT	60 mT
*Staphylococcus aureus*	10.33 ± 0.00 ^a^	8.33 ± 0. 35 b	6.66 ± 0.35 ^bc^
*Escherichia coli*	16.66 ± 1.71 ^a^	13.33 ± 1.71 ^a^	13.33 ± 1.71 ^a^
	**MBC (mg/ml)**		
	**Co**	**30mT**	**60 mT**
*Staphylococcus aureus*	16.66 ± 4.00 ^a^	13.33 ± 4.71 ^a^	10.00 ± 4.71 ^b^
*Escherichia coli*	20.00 ± 0.00 ^a^	16.66 ± 4.71 ^a^	13.33 ± 7.71 ^a^

• *Different lowercase letters indicate significant differences in each row (*P* < 0.05)

#### Inhibition of bacterial growth (OD)

According to the result presented in Table 4, a significant difference was observed between different treatments (*p* < 0.05). The results showed that *Staphylococcus aureus* was more sensitive to the nanoparticles, and a higher percentage of them were inhibited (*p* < 0.05). In the comparison between the treatments, the nanoparticles obtained in the control condition showed the least inhibition of growth on both bacteria (*p* < 0.05). The extracts obtained at 30 and 60 mT did not have a significant impact on inhibiting the growth of *Staphylococcus aureus* (*p* > 0.05). However, the results showed that increasing the intensity of the magnetic field from 30 to 60 mT showed more growth inhibition against *Escherichia coli* bacteria (*p* < 0.05). In general, *Escherichia coli* was more resistant and had a higher percentage of survivors (*p* < 0.05).

**Table 4:** Inhibition of bacterial growth (OD) of selenium nanoparticles

	Control	30 mT	60 mT
*Staphylococcus aureus*	41.02 ± 0.95 ^b^	42.82 ± 0.95 ^a^	42.82 ± 0.72 ^a^
*Escherichia coli*	37.17 ± 0.36 ^c^	39.23 ± 0.62 ^b^	40.25 ± 0.36 ^a^

•Different lowercase letters indicate significant differences in each row (*P* < 0.05).

### Results of antifungal activity of coated selenium nanoparticles

The results of the antifungal activity of coated selenium nanoparticles are presented in Table 5. According to the results, *Aspergillus niger* was more sensitive than *Penicillium chrysogenum* to *Alborzia kermanshahica* (*p* < 0.05). In the comparison, cyanobacteria grown in control conditions showed the least antifungal properties (*p* < 0.05). The highest antifungal property was also observed in the extract of cyanobacteria grown under a 60 mT magnetic field (*p* < 0.05).

**Table 5:** Inhibition zone diameter of selenium nanoparticles

	Control	30 mT	60 mT
***Aspergillus niger***	6.66 ± 0.47 ^b^	7.66 ± 0.47 ^ab^	8.00 ± 0.00 ^a^
***Penicillium chrysogenum***	7.33 ± 0.47 ^b^	8.00 ± 0.81 ^ab^	8.33 ± 0.47 ^a^

• Different lowercase letters indicate significant differences in each row (*P* < 0.05)

### The results of TEM analyze

**Fig. 5:**
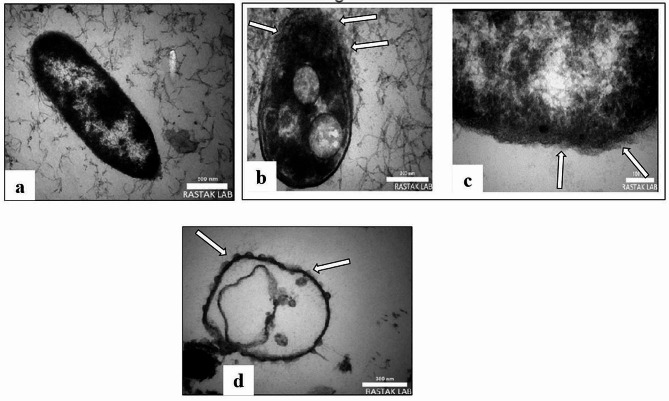
TEM photomicrographs (scale bar = 100 nm) of E. *coli* cells after treatment with SeNPs (arrows) for different incubation periods under 60 mT. (**a**) Untreated cell, (**b**) cell treated for 30 min, (**c**) cell treated for 60 min, (**d**) cell treated for 120 min

The time-dependent interaction between selenium nanoparticles and E. *coli* revealed time-dependent outer membrane alterations. TEM scanning showed the attachment of SeNPs to the outer membranes of E. *coli* (arrows) after 30 min of incubation (Fig. 5b). After 60 min of incubation, SeNPs induced degradation and rupture of the outer cell membranes; therefore, the cells start to shrinkage and appear in irregular shape compared with the control untreated cells (Fig. 5a, c). After incubation for 2 h, cell membranes were disrupted and leakage of the intracellular contents was observed (Fig. 5c). Complete loss of the cell membranes was observed as a sign of complete fragmentation after 120 min (Fig. 5d). Furthermore, TEM scanned the attachment of SeNPs on different sites of S. E. *coli* membrane (arrows) after 120 min of incubation with SeNPs. In the attachment sites, the mycelial membrane was observed to be weak, disrupted, therefore gained light staining in comparison with the control untreated membrane (Fig. 5a).

### The results of cytotoxicity test

The amount of *Alborzia kermanshahica* cyanobacterial extract used to kill Hep G-2 cancer cells directly affected the number of cells that died (Table 6). It was found that increasing the concentration of *Alborzia kermanshahica* cyanobacterial extract grown at 60 mT made it better at stopping the growth of Hep G-2 cancer cells.

**Table 6:** The cytotoxicity effect of SeNPs on Hep G-2

Concentration (ppm)	Viability (%)
0.00	100.00 ± 0.00 ^k^
0.20	99.16 ± 0.40 ^k^
0.39	97.26 ± 0.80 ^g^
0.78	95.47 ± 0.41 ^i^
1.56	94.64 ± 0.89 ^h^
3.12	85.92 ± 0.95 ^g^
6.25	82.74 ± 0.47 ^f^
12.50	77.16 ± 0.82 ^e^
25	72.87 ± 0.33 ^d^
50	61.77 ± 0.40 ^c^
100	55.28 ± 0.20 ^b^
200	54.56 ± 0.36 ^a^

• Different lowercase letters indicate significant differences in each row (*P* < 0.05)

## Discussion

The green biosynthesis of nanoparticles by cyanobacteria depends on the bottom-up technique [56]. Cyanobacteria include a variety of bioactive natural chemicals that are biocompatible reductants, such as polysaccharides, proteins, pigments, and antioxidants, making them a safe and environmentally acceptable platform for the creation of metallic nanoparticles [57].

Chitosan (CS) is a kind of alkaline polysaccharide characterized by non-toxicity, good biocompatibility, biodegradability, and low immunogenicity. Interestingly, the modification of SeNPs with CS (CS-SeNPs) can significantly improve the stability of SeNPs in an aqueous solution and antioxidant activity. As an abundant natural polysaccharide, CS can interact with SeNPs and prevent them from aggregating due to their hydrophilic groups. In a study by Chunyan Shao et al. (2022) the coated-selenium nanoparticle with chitosan was used to improve the stability of nanoparticle with the objective in the inhibitory effect of against respiratory syndrome virus (PRRSV) replication [58].

Selenium has been studied for the treatment of inflammatory diseases such as rheumatoid arthritis and asthma [59], and for cancer treatment as a chemotherapeutic or radiotherapeutic adjuvant. Compared with normal cells, malignant cells exhibit more oxidative stress; therefore, cancer cells are more susceptible to the pro-oxidant effects of selenium than normal cells [60–62].

Traditional uses of Se are risky because of toxicity and the difficulty distinguishing between Se deficiency, appropriate intake, and excess intake [63]. The use of Se-containing compounds as potential chemo-therapeutic agents is limited because high levels of Se supplementation can induce toxicity [61]. However, using the proper amount of Se provides an outstanding benefit for treatment. Since the narrow therapeutic index of Se is a concern, nanotechnology plays a crucial role in developing selenium nanoparticles (SeNPs) to overcome this obstacle by reducing toxicity and improving biocompatibility [61, 64]. SeNPs have been applied in several biomedical applications as a drug delivery system, especially as an anti-cancer agent for cancer treatment [63, 65–67]. The efficiency of SeNPs in inhibiting tumor cell growth in vitro and in vivo was demonstrated in cancers such as liver, breast, prostate, lung, and brain cancer [68–73]. Compared with the same high dose of soluble Se, which is commonly toxic, SeNPs were found to be effective and well tolerated in vivo [74, 75]. SeNPs comprise an inorganic therapeutic core of SeO that can be stabilized or functionalized in the loaded active drug or specific compounds [63, 76]. For coating or functionalizing SeNP, several approaches have been studied to improve stability and target the therapeutic effect [77]. Numerous compounds have been used to modify the surface of SeNP, such as folic acid, hyaluronic acid, chitosan, polysaccharides, amino acids, peptides, and proteins [40–44]. Therefore, this study assessed the potential of chitosan-coated selenium nanoparticles produced under magnetic field as a novel anticancer treatment strategy against hepatocellular cancer cells (Hep G-2).

The surface plasmon resonance, which depends on a number of variables including nanoparticle size, shape, interparticle distance, and the surrounding medium’s refractive index, determines the optical properties of metallic nanoparticles [78]. ƛmax is related to the plasmon absorption of nanoparticles, which shows that the cyanobacterial extract is responsible for the synthesis of selenium nanoparticles. UV-Vis spectrum studies have confirmed the biosynthesis of selenium nanoparticles from selenious acid. The synthesis of selenium nanoparticles is characterized by the color change from colorless selenium acid to ruby red (SeNPs). Several studies have shown that the colloidal solution containing SeNPs has λmax at a wavelength of 250 to 350 nm [79].

Afzal et al. (2019) reported that the color changes observed in selenium nanoparticles was for the reduction of the reaction of SeO^− 2^ to Se^0^ [80]. According to the studies of Sharma et al. (2014), the secondary metabolites found in cyanobacteria are responsible for the synthesis of selenium nanoparticles [81], while Kessi et al. (1999) proposed the enzyme reductase present in the extract of cyanobacteria as the agent of synthesis [82]. Also, the broad nature of the absorption peaks of the obtained nanoparticles indicates the polydispersity nature of the nanoparticles [83].

Alipour et al. (2021) reported 450 to 500 nm of nanoparticles produced from *Spirulina platensis* during UV-visible spectroscopy [84]. Also, Srivastava et al. (2013) reported the λmax of selenium nanoparticles synthesized from Zooglea ramera at 330 nm [85]. It has been reported that the UV spectrum made by selenium nanoparticles reduced by bacteria is different because their atomic structures and sizes are different [86]. The UV-Vis spectroscopy results of this study revealed that the all the colloidal samples had ƛmax in the reported range and the formation of nanoparticles was confirmed by cyanobacteria extract.

FT-IR is a tool that provides essential details about the chemical interactions involved in the bio reduction of Se^+ 4^ and the stabilization of SeNPs. FTIR spectroscopy results showed consistent spectra, but the increased intensity of peaks indicates an increase in bond number after exposure to 30 mT and 60 mT. The results of FTIR spectra of samples of selenium nanoparticles from other cyanobacterial strains are also cofirmed by various studies [87–91]. The results of our studies were in line with other scientists.

With respect to particle size distribution characterization, a parameter used to define the size range of the lipidic nanocarrier systems is called the “polydispersity index” (PDI). The term “polydispersity” (or “dispersity” as recommended by IUPAC) is used to describe the degree of non-uniformity of a size distribution of particles. The PDI value may vary from 0 to 1, where colloidal particles with a PDI less than 0.1 indicate monodisperse particles and values greater than 0.1 may indicate a polydisperse particle size distribution [92]. Studies have shown that when the PDI value is less than 0.3, it indicates that the degree of dispersion is good [93]. The results showed the polydispersity distribution of selenium nanoparticles prepared from cyanobacteria extract, and the nanoparticles prepared from cyanobacteria grown at different magnetic field intensities had a much more uniform dispersion than the nanoparticles prepared with control extract. Faramarzi et al. (2020) reported the PDI of selenium nanoparticles prepared from *Saccharomyces cerevisiae* yeast at a concentration of 5 to 25 µg of extract between 0.989 and 0.189. They showed that the selenium nanoparticles formed using yeast had the maximum amount of selenium salt and were more uniform in size [94]. Alipour et al. (2021), while investigating the synthesis of selenium nanoparticles from the cyanobacterium *Spirulina platentis* extract under different pH conditions, observed the size of nanoparticles in the range of 136–190 nm and the PDI of nanoparticles in the range of 4.2–17.6 [84]. Zhang et al. (2020), evaluated the synthesis of selenium nanoparticles from the cyanobacterium *Spirulina platentis* extract. They reported that the size distribution of selenium nanoparticles was 0.14 and showed that the nanoparticles were uniform [95].

Zeta potential analysis was conducted to ascertain the surface charges accumulated by selenium nanoparticles. By observing the mobility of particles when exposed to an electric field, one can determine the zeta potential. The movement of particles is contingent upon the concentration of electrolytes and the charge present on the surface. Studies have shown that a zeta potential of -30 mV is considered optimal for the stabilization of a nano dispersion [96]. Therefore, it can be said that the nanoparticles prepared by applying a magnetic field of 30 and 60 mT had optimal dispersion compared to the nanoparticles prepared from the control sample.

Filipović et al. (2020) examined the selenium nanoparticles synthesized by the chemical reduction method with three different coatings: SeNPs-BSA, SeNPs-Chit, and SeNPs-Gluc, and reported a zeta potential of + 0.27, 24, and − 45 mV, respectively [97]. Abbas and colleagues (2021), during the synthesis of selenium nanoparticles from cyanobacterial extract, reported the potential value of nanoparticles as -32.9 mV. These researchers attributed the negative zeta potential to the negative charge potential value due to reducing factors (polysaccharides and proteins) found in cyanobacteria and reported that if all nanoparticles in suspension show negative zeta potential, electrostatic repulsion between particles and high stability of nanoparticles occur [98].

Selenium nanoparticles (SeNPs) have attracted great interest as a potential antimicrobial agent. However, there is limited research on the antibacterial activity and possible mechanisms of biosynthesized SeNPs. The limited mechanism has been reported in a few references to the antibacterial properties of bio-SeNPs. Beheshti et al. (2013) reported that bio-SeNPs synthesized by *Bacillus* sp. could induce apoptosis in the protozoa Leishmania major through DNA fragmentation [99]. Besides, the addition of bio-SeNPs could effectively promote the production of reactive oxygen species (ROS), but their intensity did not show a size- or shape-dependent effect [100]. And other unknown mechanisms still need to be discovered. However, the antimicrobial activity of other nanoparticles was generally achieved by the damage to mitochondria, disruption of cell membranes, and even interrupting transmembrane electron transport [101].

The result of inhibition zone diameter showed that no significant difference was observed between magnetic fields of 30 and 60 mT. However, the *Staphylococcus aureus* strain showed more sensitiveness compared to the *Escherichia coli* strain. The cause of this has been reported to be the permeability layer of the outer membrane in Gram-negative bacteria [102]. Pandey et al. (2021) investigated the antibacterial effect of selenium nanoparticles prepared from cyanobacterial extract against two bacteria, *Staphylococcus aureus* and *Escherichia coli*. They showed that *Staphylococcus aureus* bacteria were more sensitive than *Escherichia coli* bacteria. They also reported the inhibition zone of *Staphylococcus aureus* as 11 mm [103]. Rangrazi et al. (2020) evaluated the antibacterial effects of selenium nanoparticles in chitosan coating on gram-positive and gram-negative bacteria. They revealed that gram-positive bacteria were more sensitive to nanoparticles. They showed that nanoparticles did not show a significant antibacterial effect against Escherichia coli Gram-negative bacteria [104]. It has been suggested that the antimicrobial activity of nanoparticles may be due to the release of ions in the environment where microorganisms grow. It has also been found that the dissolution of ions depends on the size of nanoparticles and the concentration of the solution [105]. Research showed that selenium nanoparticles have size- and concentration-dependent antimicrobial effects against various microorganisms [106]. In another study conducted by Adibian et al. (2022), the MIC levels of selenium nanoparticles synthesized by the green method against *Mycobacterium tuberculosis*, *Staphylococcus aureus*, *Streptococcus mutans*, *Escherichia coli*, and *Pseudomonas aeruginosa* were 256, 16, 32, 128, and 64 µg/mL, respectively [107].

The MIC and MBC analyses were also performed in this research. The result showed that the application of 60 mT magnetic fields had the same inhibitory effect on *Staphylococcus aureus* and *Escherichia coli* compared to the control sample. Based on the results of bacterial growth inhibition, increasing the intensity of the magnetic field from 30 to 60 mT caused more growth inhibition against *Escherichia coli* bacteria (*p* < 0.05). In general, *Escherichia coli* was more resistant and had a higher percentage of survivors (*p* < 0.05).

Many have been devoted to the antifungal effect of selenium nanoparticles; however, this effect is less than the antibacterial effect. In most fungal studies, the following species are used: *Candida* [108–114] and *Fusarium* [112, 115–117]. Apart from these, the following fungal species are used: *Colletotrichum* [116, 118], *Puccinia* [119], *Aspergillus* [120, 121], *Cryptococcus* [109], *Penicillium* [109], *Rhizoctonia* [122], *Pyricularia*, and *Alternaria* [118]. Antifungal mechanisms include antibiofilm activity [97, 123], ROS generation and oxidative stress (with the addition of the antifungal drug ketoconazole) [124] and influence on the expression of fungicidal drug resistance genes [125].

The results of the antifungal activity of nanoparticles produced under 60 mT showed the highest activity. In the comparison between the extracts obtained, it was found that the extract of cyanobacteria grown in control conditions showed the least anti-fungal properties compared to the magnetic fields of 30 and 60 mT. In line with the results of this research, Lazcano-Ramírez et al. (2023) evaluated the antifungal properties of selenium nanoparticles synthesized by the green method in concentrations of 0, 0.25, 0.5, 1, and 1.7 mg/ml. The results showed that selenium nanoparticles had strong antifungal activity against *Fusarium oxysporum* and *Colletotrichum gloeosporioides* [116]. Joshi et al. (2021) investigated the synthesis of selenium nanoparticles from *Trichoderma atroviride* extract and observed that, at a concentration of 100 ppm, the use of nanoparticles had a good inhibitory effect against the fungus *Pyricularia grisea* [126].

To understand the antibacterial mechanism of SeNPs on Gram-negative bacteria, E. *coli* was chosen as a model to study the effect of SeNPs on the permeability and membrane structure of E. *coli* cells. The result showed that SeNPs-treated membrane vesicles were dissolved and dispersed, and their membrane components were disrupted and dispersed from their original order and close arrangement. The cell membrane broke down and lost its intrinsic function. TEM micrographs of SNP-treated E. coli cells showed that large gaps appeared in the cell membrane, and the bacteria were almost completely disintegrated into several parts [127].

It is well known that gram-negative bacteria have an outer membrane outside the peptidoglycan layer, which gram-positive bacteria do not have. The main function of the outer membrane is to act as a selective permeability barrier and protect bacteria from harmful agents such as detergents, drugs, toxins, and degrading enzymes while providing nutrients to sustain bacterial growth. The structure and chemical composition of the outer membrane in E. *coli* cells have been widely studied. The outer membrane has an uneven lipid bilayer with an inner leaflet mostly made up of phospholipid chains and an outer leaflet mostly made up of lipopolysaccharide molecules. It has been estimated that approximately 3.5 million lipopolysaccharide molecules cover three-quarters of the E. *coli* surface, and the remaining quarter is composed of membrane proteins. Evidence from genetic and chemical experiments has proven that the lipopolysaccharide layer of the outer membrane plays an essential role in creating a selective permeability barrier for E. *coli* and other Gram-negative bacteria, and the modified lipopolysaccharide structures of the mutation found that they can increase permeability compared to native cells [128].

Studies have shown that SeNPs apparently increase membrane permeability to reduce sugars and proteins in cells. Therefore, it can be stated that the disruption of membrane permeability will be an important factor in inhibiting the growth of bacteria. But it is still a mystery which part of the lipopolysaccharide or membrane proteins in the outer membrane is damaged. In addition, the outcomes of numerous studies have demonstrated that SeNPs may inhibit the activity of respiratory chain dehydrogenases in E. *coli*. The higher the concentration of SeNPs, the lower the enzyme activity. It is assumed that SeNPs may cross the permeability barrier of the outer membrane, peptidoglycan, and periplasm, destroy dehydrogenases of the respiratory chain, and also inhibit cell respiration [129]. These phenomena indicate possible antibacterial mechanisms through which SNPs inhibit bacterial growth, as well as cellular responses to SeNPs treatment.

Based on the current research, the action model of SeNPs can be described as SeNPs that break the permeability of the outer membrane and lead to the leakage of cellular materials. Secondly, SeNPs enter the inner membrane and deactivate dehydrogenases of the respiratory chain, thus inhibiting the respiration and growth of cells. At the same time, SeNPs can affect some proteins and lipid phosphates and cause membrane collapse, which leads to cell disintegration and eventually death [130]. In line with the results of our study, Li et al. (2010) showed that silver nanoparticles are able to destroy the permeability of bacterial membranes [130]. When the E. *coli* cells were exposed to the carrier, many holes and gaps were observed in the bacterial cells with a scanning electron microscope, and the cell membrane was fragmented, which shows that the bacterial cells are severely damaged and their membrane components were scattered from their original orderly arrangement based on TEM observations.

The results of the cytotoxicity test showed that nanoparticles produced under 60 mT had the highest inhibition percentage against Hep G-2 cancer cells. Similar to our results, Wadhwani et al. (2017) investigated the anticancer properties of nanoparticles synthesized by *Acinetobacter* sp. They showed that selenium nanoparticles had anti-proliferative activity against breast cancer cells, which was dependent on the dose of nanoparticles used [131]. Hashem et al. (2021) showed the anticancer property of selenium nanoparticles synthesized from bacterial extract, and the reason for this was the release of SeNPs to the cell membrane through ion channels and contact with DNA or protein nitrogenous bases. Intracellular factors have been explained to cause cell cycle arrest, mitochondrial dysfunction, DNA fragmentation, and cell apoptosis [132].

## Conclusion

Nanoparticles have significant inhibitory effects on numerous cancer cells due to their advanced properties. Meanwhile, the combined use of nanoparticle synthesis and the use of magnetic fields to destroy cancer cells can replace many chemical anticancer drugs. In addition, coating nanoparticles with chitosan not only leads to their stability, but also has much less toxicity compared to other natural polysaccharides and has very wide applications in biomedicine. The use of this technology can be considered an important step in introducing the cytotoxicity effects of nanoparticles produced by using cyanobacterial strains, which can lead to the treatment of many cancer diseases. For this reason, investigating the anticancer activity of nanoparticles synthesized by cyanobacteria and simultaneously using the magnetic fields can be a novel strategy as an anticancer treatment. As magnetic fields can add new dimensions to nanoparticle applications. Therefore, this is a first attempt towards biosynthesis of SeNPs using *Alborzia kermanshahica* cultivated under different magnetic fields of 30 mT and 60 mT. The results of this study revealed that production of selenium nanoparticles by using *Alborzia kermanshahica* cultivated under magnetic field of 60 mT showed a great toxicity, antibacterial and antifungal activity.

## Data Availability

All data generated or analysed during this study are included in this published article.

## Declarations

### Live vertebrates and/or higher invertebrates

this study is not on live vertebrates and/or higher invertebrates.

### Images

all the images are original and not copy from other papers.
